# Estimating the viscosity of volcanic melts from the vibrational properties of their parental glasses

**DOI:** 10.1038/s41598-021-92407-5

**Published:** 2021-06-22

**Authors:** Michele Cassetta, Danilo Di Genova, Marco Zanatta, Tiziana Boffa Ballaran, Alexander Kurnosov, Marco Giarola, Gino Mariotto

**Affiliations:** 1 Dipartimento di Informatica, Università di Verona, 37134 Verona, Italy; 2 BGI, Bavarian Research Institute of Experimental Geochemistry and Geophysics, University of Bayreuth, 95447 Bayreuth, Germany; 3 Dipartimento di Fisica, Università di Trento, 38123 Trento, Italy; 4 CPT, Centro Piattaforme Tecnologiche, Università di Verona, 37134 Verona, Italy

**Keywords:** Raman spectroscopy, Volcanology, Glasses, Rheology, Petrology

## Abstract

The numerical modelling of magma transport and volcanic eruptions requires accurate knowledge of the viscosity of magmatic liquids as a function of temperature and melt composition. However, there is growing evidence that volcanic melts can be prone to nanoscale modification and crystallization before and during viscosity measurements. This challenges the possibility of being able to quantify the crystal-free melt phase contribution to the measured viscosity. In an effort to establish an alternative route to derive the viscosity of volcanic melts based on the vibrational properties of their parental glasses, we have subjected volcanologically relevant anhydrous glasses to Brillouin and Raman spectroscopic analyses at ambient conditions. Here, we find that the ratio between bulk and shear moduli and the boson peak position embed the melt fragility. We show that these quantities allow an accurate estimation of volcanic melts at eruptive conditions, without the need for viscosity measurements. An extensive review of the literature data confirms that our result also holds for hydrous systems; this study thus provides fertile ground on which to develop new studies of the nanoscale dynamics of natural melts and its impact on the style of volcanic eruptions.

## Introduction

Volcanic activity encompasses the slow extrusions of lava domes, relatively calm emission of lava flows, as well as the extremely fast and dramatic injection of volcanic ash and gas into the atmosphere and deposition of fast-moving density currents on the Earth’s surface. The study of the mechanisms that determine the timescale and style of eruptions is central for the assessment of natural hazards and risk mitigation^1,2^.

Both the timescale and style of volcanic eruptions largely depend on a complex interplay between the physicochemical properties of magmas and processes that occur during their ascent to the surface^3,4^. Macroscopically, the flow of volcanic melts and their viscoelastic response to deformation are dominantly controlled by the shear viscosity ${\eta }_{s}$^5,6^. The shear viscosity also affects microscale processes such as the kinetics of nucleation and growth of crystals and gas phases as well as their separation from the carrying liquid phase^3,7,8^. The interaction and timescale of these processes ultimately impact the magma ascent pathway, thus concurring to determine the dynamics, fate and style of volcanic eruptions. Consequently, accurate knowledge of ${\eta }_{s}$ is pivotal to simulate the expected eruptive scenarios of a given volcano and assess the associated risks^9^.

The determination of the shear viscosity of volcanic melts is intrinsically based on viscosity data measured as a function of the liquid temperature $T$ and chemical composition ${\chi }$. High-quality viscosity data are essential to accurately parameterize ${\eta }_{s}\left(T,\chi \right)$ because small changes in ${\chi }$ in specific chemical domains can affect ${\eta }_{s}$ by several orders of magnitude^10–16^. Concentric cylinder viscosimetry and falling sphere measurements are central to measure the low viscosities (${\eta }_{s}<$ 10^5^ Pa s) of anhydrous (i.e. volatile-free) and volatile-bearing (mainly H_2_O, CO_2_ and S) melts at eruptive temperatures (from ~ 800 °C for rhyolites to ~ 1200 °C for basalts). However, concentric cylinder viscosimetry requires large quantities of material (~ 100 g) and can only measure the anhydrous viscosity because it operates at ambient pressure, whereas falling-sphere measurements of volatile-bearing samples are experimentally challenging, time consuming and require expensive high-pressure and high-temperature equipment. In contrast, micropenetration and parallel plate measurements, which operate at ambient pressure and require a few mg of doubly polished glass with a thickness of ideally ~ 3 mm, are routinely applied to measure the high viscosities (${\eta }_{s}>$ 10^9^ Pa s) of volatile-free and -bearing melts. These measurements are carried out at temperatures well below those relevant to volcanic processes for most magmas, around and slightly above the glass transition temperature ${T}_{g}$ [${\eta }_{s}\left({T}_{g}\right)=$ 10^12^ Pa s], where crystallisation or volatile exsolution is commonly assumed to be delayed relative to the measurement timescale. However, experimental observations have recently reported that micro and nanoscale modifications (i.e. crystallization and/or phase separation) can occur within minutes when glasses are heated above their ${T}_{g}$ in the supercooled liquid region, or even within seconds at shallow undercooling when melts are subjected to fast cooling rates^17–22^. These modifications dominantly result in the formation of iron-bearing nanocrystals that increase the shear viscosity through complex chemical and physical mechanisms, such as the chemical modification of the melt, formation of high-viscosity shells around crystals, and possible formation of aggregates that effectively increase the solid volume fraction in the melt^17,21,23^. An alternative experimental approach to estimate the viscosity of anhydrous and hydrous melts is based on differential scanning calorimetry (DSC) measurements (Ref.^24^ and references therein). This method effectively minimizes or even avoids the impact of melt heat treatment and requires small (~ 10 mg) quantities of material. However, DSC-derived viscosities are limited to viscosity intervals around ${T}_{g}$ (10^10^ $\lesssim {\eta }_{s}\lesssim$ 10^12^ Pa s), whereas volcanic melts mostly erupt at temperatures hundreds of degrees higher. As such, the extrapolation of the measured viscosity of natural melts around ${T}_{g}$ to volcanologically relevant, higher temperatures is essential and required.

Several theoretically and empirically based models (Ref.^25^ and references therein) describe the temperature dependence of the viscosity of glass-forming liquids. A particularly effective approach is the Mauro–Yue–Ellison–Gupta–Allan (MYEGA) formulation^25^ that describes melt viscosity from the knowledge of three key parameters: melt fragility, $m\left({\chi }\right)$; glass transition temperature, ${T}_{g}\left({\chi }\right)$ and viscosity at infinite temperature, ${\eta }_{\infty }\left({\chi }\right)$. The temperature dependence of the shear viscosity ${\eta }_{s}$ is described according to:

1 $${\text{log}}_{10}{\eta }_{s}\left(T\right)={\text{log}}_{10}{\eta }_{\infty }+\left(12-{\text{log}}_{10}{\eta }_{\infty }\right)\frac{{T}_{g}}{T}\text{exp}\left[\left(\frac{m}{12-{\text{log}}_{10}{\eta }_{\infty }}-1\right)\left(\frac{{T}_{g}}{T}-1\right)\right]$$

where $m$ is the melt fragility^26^:

2 $$m={\left.\frac{\partial {\text{log}}_{10}{\eta }_{s}}{\partial {T}_{g}/T}\right|}_{T={T}_{g}}$$

The melt fragility $m$ describes how fast the melt viscosity increases when ${T}_{g}$ is approached upon cooling. Glass-forming melts are classified as “strong” or “fragile” according to the value of this parameter. Strong melts have low $m$ values and are characterized by a quasi- or purely-Arrhenius dependence when ${\text{log}}_{10}{\eta }_{s}$ is plotted against ${T}_{g}/T$ in the so-called Angell plot^26^, whereas fragile liquids exhibit non-Arrhenius behaviour in the same plot and are characterized by high $m$ values.

Of particular interest here are the glass formation process and possibility that some melt properties can be embedded in its glass. Glass formation takes place upon the cooling of a melt into a supercooled liquid (SCL) state—down below its ${T}_{g}$—at a sufficiently fast rate to avoid crystallization. This process is rooted in the timescale dependence of the structural relaxation ${\tau }$ of the melt with temperature. During cooling τ increases exponentially, exceeds the laboratory (experimental) timescale and the metastable equilibrium rearrangement of atoms can thus not take place. Because the rearrangement of atoms is virtually hampered within the observational timescale, the melt properties deviate from the metastable equilibrium and the melt structure is frozen into the glassy state; a nonequilibrium, non-crystalline condensed state of matter whose structure is similar to that of its SCL^27^. The glass spontaneously relaxes toward the SCL state, but this occurs over an infinitely long time if the glass is not subjected to certain thermal treatment. Nevertheless, the glass structure can be probed to retrieve fundamental information regarding the melt properties. This seems counterintuitive because glass properties are expected to depend on the cooling rate to which the liquid is subjected.

However, in their seminal work, Scopigno et al.^28^ observed and correlated the vibrational properties of glass well below ${T}_{g}$ with $m$. In particular, they showed a correlation between the melt fragility and temperature behaviour of the nonergodicity factor obtained through inelastic X-ray scattering experiments. The nonergodicity factor is defined as the long time limit of the density–density correlator and increases from zero in the liquid to a finite positive value when the system enters into the glassy state and atoms are frozen into a disordered liquid-like structure^29,30^. The only possible decorrelation is given by the vibrations, which become progressively suppressed upon further temperature reduction while the nonergodicity factor increases. The parameter that controls this temperature behaviour is proportional to $m$, thus suggesting that the melt fragility $m$ is embedded in the vibrational properties of the resulting glass. Novikov and Sokolov^31^ later explored the relationship between the melt fragility and instantaneous bulk (${K}_{{\infty }}$) and shear (${G}_{{\infty }}$) elastic moduli of various non-metallic glasses. The elastic moduli were obtained from the longitudinal (${v}_{p}$) and shear (${v}_{s}$) sound velocities measured by Brillouin light scattering (BLS) and the results showed a linear correlation between ${K}_{{\infty }}/{G}_{{\infty }}$ and *m*. However, the correlation fails if extended to a larger data-set of glasses (i.e. metallic, polymeric, chalcogenide and organic systems) resulting in a not-universal law^32^. It has been proposed that the free-electron contribution to the bulk modulus of metals and a specific intramolecular contribution to the fragility in the case of polymers for some long molecules are the main reasons for the failure of the correlation^33^. Furthermore, Novikov and Sokolov^31^ showed that $m$ linearly correlates with the inverse amplitude of the boson peak (BP). Examining the vibrational density of state of glasses in a $g\left(\omega \right){/\omega }^{2}$ plot, this spectral feature can be identified as the broad low-frequency maximum exceeding the ${\omega }^{2}$ prediction according to the Debye model. The origin of the BP is still an open question (Ref.^34^ and references therein) and has been largely studied by means of several experimental techniques including Raman spectroscopy (RS).

These intriguing observations suggest that the study of the vibrational properties of glasses has the potential to derive $m\left({\chi }\right)$ and thereby allow the parameterization of melt viscosity at any temperature without the need to measure the viscosity. This can be critical for volcanic liquids that tend to undergo modifications during viscosity measurements. Moreover, volcanic glass is often embedded in rocks containing crystals, which does not allow direct liquid viscosity measurements. Here, we test and further explore the literature hypotheses^28,31^ by subjecting simple and multicomponent glasses to Brillouin and Raman spectroscopic measurements. We explore a chemical space of dry and hydrous glasses that virtually encompass the full range of magmatism on Earth. Samples were characterized using both Brillouin and Raman spectroscopy and the results show that the fragility $m$ correlates with both the $K/G$ determined by BLS and boson peak position obtained by RS. The results from our study demonstrate that the viscosity of volcanic melts at eruptive temperatures can be accurately predicted from the spectroscopic analysis of glass. The proposed approach was carefully validated using external samples from the literature for which the viscosity is known and Brillouin and Raman data are available. This opens a new scenario for modelling the chemical contribution to the viscosity of glass-forming melts on a physically substantiated basis using their parental glasses.

## Results

Table 1 lists the chemical composition of the glasses considered in this study. We measured the vibrational properties of 16 anhydrous samples (from RhA to Di) using BLS and RS, whereas data of 11 anhydrous samples (from SiO_2_ to HPG8_Li05) were collected from the literature (see references in Table 1). An additional set of 27 samples [anhydrous (6) and hydrous (21), Table 2)] was included in the dataset for the external validation of our approach.

**Table 1:** Chemical composition (wt%) of samples used and considered in this study.

	SiO_2_	TiO_2_	Al_2_O_3_	FeO_(t)_	MnO	MgO	CaO	Na_2_O	K_2_O	Fe_2_O_3_/GeO_2_/Li_2_O/P_2_O_5_/SO_3_	References
RhA	77.63	0.11	12.73	3.03	0.03	0.06	0.92	4.44	1.62	–	^10^
RhB	77.28	0.14	13.39	2.94	0.02	0.06	0.75	2.71	3.61	0.03 (P_2_O_5_)	^10^
RhD	76.83	0.11	12.43	2.96	0.05	0.07	0.9	2.93	4.29	0.04 (P_2_O_5_)	^10^
RhE	75.33	0.12	13.61	2.93	0.03	0.07	0.88	1.41	6.80	0.06 (P_2_O_5_)	^10^
RhG	77.86	0.12	11.69	2.99	0.04	0.07	0.85	1.05	5.34	–	^10^
RhH	77.25	0.10	11.95	2.62	0.06	0.22	1.08	3.23	4.35	0.05 (P_2_O_5_)	^10^
RhI	76.24	0.06	11.46	2.85	0.03	0.44	1.31	2.99	3.83	0.04 (P_2_O_5_)	^10^
RhJ	73.75	0.31	11.99	3.31	0.07	1.64	2.98	3.16	3.56	0.02 (P_2_O_5_)	^10^
MSA	59.58	0.58	17.94	6.28	0.20	2.86	7.71	3.75	0.84	–	^37^
HO	66.17	0.77	15.96	5.02	0.12	1.70	4.65	3.70	2.23	0.1 (P_2_O_5_)	^38^
Str	49.30	0.86	16.90	8.09	0.16	6.12	12.00	2.74	2.14	0.5 (P_2_O_5_)	^a^
Etn	48.99	1.70	17.05	10.11	0.25	5.57	10.22	3.75	1.87	–	^20^
DGG-1	71.72	0.14	1.23	–	–	4.18	6.73	14.95	0.38	0.19 (Fe_2_O_3_); 0.44 (SO_3_)	^20^
An	43.19	–	36.65	–	–		20.16	–	–	–	^20^
Crd	52.26	–	34.66	–	–	12.90	0.17	–	–	–	^20^
Di	55.35	–	–	–	–	18.46	26.19	–	–	–	^20^
SiO_2_	100	–	–	–	–	–	–	–	–	–	^39,46^
GeO_2_	–	–	–	–	–	–	–	–	–	100 (GeO_2_)	^42,47,48^
And	62.40	0.55	20.01	–	0.02	3.22	9.08	3.52	0.93	–	^17^
Bas	50.22	2.63	18.91	–	–	11.15	12.46	3.19	1.40	–	^35^
Phon	58.82	0.79	19.42	–	–	1.87	2.35	9.31	7.44	–	^35^
Trach	64.45	0.50	16.71	–	–	2.92	5.36	6.70	3.37	–	^35^
Teph	50.56	2.35	14.03	–	–	8.79	15.00	7.04	3.01	–	^35^
Foid	43.57	2.97	10.18	–	–	9.17	26.07	7.59	0.96	–	^35^
HPG8_Na05	74.10	–	11.70	–	–	–	–	9.00	4.40	–	^41^
HPG8_K05	74.60	–	11.80	–	–	–	–	4.40	9.20	–	^41^
HPG8_Li05	73.20	–	12.90	–	–	–	–	4.3	4.40	4.9 (Li_2_O)	^41^

^a^This study.

**Table 2:** Brillouin and Raman data, as well as MYEGA parameters (*m* and *T*_*g*_) from this study and the literature.

Sample	*v*_*S*_ (m s^−1^)	*v*_*P*_ (m s^−1^)	*K/G*	*ω*_*BP*_ (cm^−1^)	*m*^MYEGA^	*m*^BLS;RS^	*T*_*g*_ (K)	References
RhA	3604 (13)	5835 (12)	1.29 (0.02)	53.2 (0.5)	25.3 (0.1)	26.2; 25.1	1043.8 (1.1)	(*η*)^10^
RhB	3610 (8)	5869 (14)	1.28 (0.02)	49.1 (0.4)	27.1 (0.4)	26.1; 24.6	1093.0 (3.5)	(*η*)^10^
RhD	3595 (8)	5810 (20)	1.28 (0.02)	51.2 (0.5)	27.2 (0.3)	24.7; 25.3	1097.4 (2.4)	(*η*)^10^
RhE	3550 (14)	5773 (11)	1.31 (0.02)	47.8 (0.3)	26.9 (0.2)	26.1; 24.2	1108.0 (2.5)	(*η*)^10^
RhG	–	–	–	48.8 (0.4)	27.5 (0.2)	24.5	1121.2 (2.0)	(*η*)^10^
RhH	3590 (11)	5803 (6)	1.28 (0.02)	54.1(0.4)	28.5 (0.2)	24.7; 26.6	1071.6 (1.5)	(*η*)^10^
RhI	3590 (14)	5820 (9)	1.30 (0.02)	54.7 (0.5)	24.9 (0.1)	25.4; 24.8	1022.0 (1.5)	(*η*)^10^
RhJ	3581 (7)	5870 (8)	1.35 (0.01)	59.2 (0.5)	26.9 (0.1)	28.0; 29.0	992.1 (0.6)	(*η*)^10^
MSA	3608 (12)	6150 (16)	1.57 (0.02)	66.5 (0.5)	33.0 (0.2)	37.4; 33.2	958.2 (0.6)	(*η*)^49^
HO	–	–	–	60.0 (0.6)	–	29.3	–	–
Str	3569 (12)	6249 (17)	1.73 (0.03)	76.0 (0.5)	40.9 (0.2)	44.4; 40.9	932.6 (1.0)	(*η*)^50,51^
Etn	–	–	–	78.2 (0.6)	–	43.1	914.2^DSC^	(*T*_*g*_)^20^
DGG-1	3451 (13)	5900 (8)	1.49 (0.02)	70.6 (0.9)	33.3 (0.6)	33.9; 36.1	813.0 (3)	(*η*)^20^
An	3753 (15)	6656 (9)	1.81 (0.03)	85.0 (0.5)	52.3 (0.4)	47.8; 51.0	1128.9 (0.6)	(*η*)^20,46,52,53^
Crd	–	–	–	88.0 (1.0)	46.6 (0.2)	55.1	1087.3 (0.2)	(*η*)^20,54^
Di	3741 (15)	6727 (4)	1.90 (0.03)	90.0 (0.5)	55.5 (0.4)	51.6; 58.2	993.7 (0.5)	(*η*)^20,54–58^
SiO_2_	3769 (13)	5972 (20)	1.18 (0.03)	48.5 (0.5)	24.0 (0.4)	20.3; 24.4	1427.5 (7.5)	(*v*_*S*_, *v*_*P*_*,* *ω*_*BP*_)^39^, (*η*)^46^
GeO_2_	2360	3770	1.22	40.5 (0.5)	20.0 (0.2)	22.1; 22.0	816.6 (0.9)	(*v*_*S*_, *v*_*P*_)^36^, (*ω*_*BP*_)^42^, (*η*)^47,48^
And	3700	6240	1.51	–	36.2 (0.1)	34.8	1016.4 (0.3)	(*v*_*S*_, *v*_*P*_)^40^, (*η*)^17^
And (0.3)^a^	3730	6250	1.47	–	–	33.2	954.4	(*v*_*S*_, *v*_*P*_)^40^, (*T*_*g*_)^VFT^, (*η*)^17^
And (1)^a^	3750	6230	1.43	–	–	31.1	869.3	(*v*_*S*_, *v*_*P*_)^40^, (*T*_*g*_)^VFT^, (*η*)^17^
And (2.7)^a^	3550	6060	1.58	–	–	37.8	756.9	(*v*_*S*_, *v*_*P*_)^40^, (*T*_*g*_)^VFT^, (*η*)^17^
And (3.5)^a^	3620	6090	1.5	–	–	34.2	734.4	(*v*_*S*_, *v*_*P*_)^40^, (*T*_*g*_)^VFT^, (*η*)^17^
Bas	3711	6505	1.74	–	44.8 (0.1)	44.7	983.3 (0.3)	(*v*_*S*_, *v*_*P*_)^35^, (*η*)^59,60^
Bas (3.02)^a^	3586	6230	1.68	–	–	42.2	777	(*v*_*S*_, *v*_*P*_)^35^, (*T*_*g*_)^35^, (*η*)^60^
Phon	3493	5839	1.46	–	28.4 (0.2)	32.6	917.7 (0.8)	(*v*_*S*_, *v*_*P*_)^35^, (*η*)^11^
Phon (0.88)^a^	3482	5839	1.48	–	–	33.4	827	(*v*_*S*_, *v*_*P*_)^35^, (*T*_*g*_)^11^, (*η*)^11^
Phon (2.15)^a^	3394	5752	1.54	–	–	36	684	(*v*_*S*_, *v*_*P*_)^35^, (*T*_*g*_)^11^, (*η*)^11^
Phon (2.83)^a^	3372	5692	1.52	–	–	34	658	(*v*_*S*_, *v*_*P*_)^35^, (*T*_*g*_)^11^, (*η*)^11^
Phon (4.72)^a^	3372	5768	1.59	–	–	38.3	596	(*v*_*S*_, *v*_*P*_)^35^, (*T*_*g*_)^11^, (*η*)^11^
Trach	3580	5932	1.41	–	31.1 (0.1)	30.5	969.8 (0.6)	(*v*_*S*_, *v*_*P*_)^35^, (*η*)^11^
Trach (0.57)^a^	3427	5725	1.46	–	–	32.4	883	(*v*_*S*_, *v*_*P*_)^35^, (*T*_*g*_)^11^, (*η*)^11^
Trach (0.83)^a^	3416	5741	1.49	–	–	33.9	838	(*v*_*S*_, *v*_*P*_)^35^, (*T*_*g*_)^11^, (*η*)^11^
Trach (1.19)^a^	3394	5708	1.5	–	–	34.1	800	(*v*_*S*_, *v*_*P*_)^35^, (*T*_*g*_)^11^, (*η*)^11^
Trach (2.19)^a^	3351	5664	1.52	–	–	35.3	733	(*v*_*S*_, *v*_*P*_)^35^, (*T*_*g*_)^11^, (*η*)^11^
Trach (2.90)^a^	3373	5681	1.5	–	–	34.4	698	(*v*_*S*_, *v*_*P*_)^35^, (*T*_*g*_)^11^, (*η*)^11^
Trach (4.92)^a^	3329	5654	1.55	–	–	36.5	628	(*v*_*S*_, *v*_*P*_)^35^, (*T*_*g*_)^11^, (*η*)^11^
Teph	3558	6232	1.73	–	45.2 (0.8)	44.5	932.8	(*v*_*S*_, *v*_*P*_)^35^, (*T*_*g*_)^VFT^, (*η*)^61^
Teph (0.92)^a^	3580	6260	1.72	–	–	44	842.4	(*v*_*S*_, *v*_*P*_)^35^, (*T*_*g*_)^VFT^, (*η*)^61^
Teph (1.60)^a^	3591	6282	1.73	–	–	44.1	814.1	(*v*_*S*_, *v*_*P*_)^35^, (*T*_*g*_)^VFT^, (*η*)^61^
Teph (2.27)^a^	3591	6243	1.69	–	–	42.5	793.8	(*v*_*S*_, *v*_*P*_)^35^, (*T*_*g*_)^VFT^, (*η*)^61^
Foid	3525	6297	1.86	–	49.5 (0.2)	49.8	915.8 (0.2)	(*v*_*S*_, *v*_*P*_)^35^, (*η*)^61^
Foid (1.00)^a^	3525	6254	1.81	–	–	47.9	822.1	(*v*_*S*_, *v*_*P*_)^35^, (*T*_*g*_)^VFT^, (*η*)^61^
Foid (1.35)^a^	3558	6308	1.81	–	–	47.7	806.4	(*v*_*S*_, *v*_*P*_)^35^, (*T*_*g*_)^VFT^, (*η*)^61^
Foid (1.88)^a^	3514	6265	1.85	–	–	49.3	769	(*v*_*S*_, *v*_*P*_)^35^, (*T*_*g*_)^VFT^, (*η*)^61^
HPG8_Na05	3560	5790	1.31	–	23.2 (0.1)	26.1	854.6 (1.4)	(*v*_*S*_, *v*_*P*_)^41^, (*η*)^62^
HPG8_K05	3540	5721	1.28	–	22.5 (0.1)	24.7	888.8 (1.0)	(*v*_*S*_, *v*_*P*_)^41^, (*η*)^62^
HPG8_Li05	3690	6030	1.34	–	25.3 (0.2)	27.2	773.0 (1.3)	(*v*_*S*_, *v*_*P*_)^41^, (*η*)^62^

Acoustic wave velocities (*v*_*s*_ and *v*_*p*_), elastic moduli ratios (*K/G*,

Eq. 3) and boson peak positions (*ω*_*BP*_). The Brillouin and Raman spectroscopic measurements are from this study for samples RhA to Di. *m*^BLS^ is calculated using the Brillouin data (*K/G*) and Eq. (6); *m*^RS^ is calculated using the Raman data (*ω*_*BP*_) and Eq. (7); *m*^MYEGA^ and *T*_*g*_ (unless specified in the References column) are derived using Eq. (1) and viscosity data from the literature (assuming ${\eta }_{\infty }={10}^{-2.93}$ Pa s); *T*_*g*_^VFT^ is the calculated glass transition temperature using VFT parameters provided by the literature; ^DSC^ is the glass transition temperature derived via DSC in Ref.^20^ for Etn; ^a^H_2_O content in wt%. External samples used for validation (Figs. 4, 6) are RhB, And (hydrous), Bas (hydrous), Teph (dry and hydrous), Trach (dry and hydrous), Phon (hydrous), Foid (hydrous) and HPFS 7980 fused silica^63^ for the BLS approach, and RhB, MSA and Str (all anhydrous) for the RS approach.

The measured sound velocities are listed in Table 2, together with those from the literature. The longitudinal sound velocity increases with decreasing SiO_2_ content (Table 2), namely the calcalkaline rhyolites exhibit the lowest ${v}_{p}$ (between 5773 and 5870 m s^−1^), whereas the highest velocity (6727 m s^−1^) was measured for diopside (Di). The shear velocity (${v}_{s}$) does not show a significant dependence on the chemical composition and ranges between 3451 m s^−1^ for standard DGG-1 glass and 3753 m s^−1^ for anorthite (An). Our results are in line with those from Whittington et al.^35^ who studied similar FeO-free melts and found that SiO_2_-rich albite exhibited the lowest ${v}_{p}$ (5533 m s^−1^), whereas SiO_2_-poor basalt was characterized by the highest longitudinal velocity (6505 m s^−1^).

The $K/G$ ratio is calculated from the measured ${v}_{p}$ and ${v}_{s}$ as follows^36^:

3 $$\frac{K}{G}={\left(\frac{{v}_{p}}{{v}_{s}}\right)}^{2}-\frac{4}{3}$$

**Figure 1:**
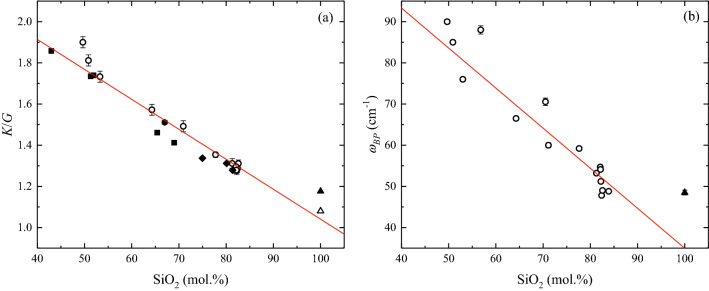
Spectroscopic parameters as a function of SiO_2_ content (mol%) of glass. Brillouin (**a**) and Raman (**b**) data of samples (Table 2) from this study (empty circles) and the literature (solid symbols) as a function the SiO_2_ (mol%) content of the glasses. The ratio of the bulk (*K*) and shear (*G*) elastic moduli is calculated (Eq. 3) from the Brillouin velocities (Table 2), whereas the boson peak position ${\omega }_{BP}$ is retrieved by fitting the low-frequency Raman spectra of the glasses using a log–normal function (see “Materials, literature data and methods” for details). Red lines in (**a**) and (**b**) are linear fits of the data with Eqs. (4, 5), respectively. Literature data in (**a**) are: (squares) Whittington et al.^35^, (hexagon) Richet and Polian^40^, (diamonds) Hushur et al.^41^, (filled triangle) Zanatta et al.^39^, (unfilled triangle) Novikov et al.^36^. Literature datum in (**b**) is from Zanatta et al.^39^.

Because BLS probes elastic properties at frequencies much higher than the structural relaxation time, we assume that the derived $K/G$ value approaches the ratio of the instantaneous ${K}_{{\infty }}$ and ${G}_{{\infty }}$ values^31,36^. Table 2 lists the calculated $K/G$ for samples subjected to BLS measurements here^10,20,37,38^ and elsewhere^35,36,39–41^, which are shown in Fig. 1a as a function of SiO_2_ in mol%. We use the SiO_2_ content (mol%) as a chemical proxy to plot $K/G$ in Fig. 1a because the samples considered in this study are silicate glasses. However, in the discussion section, we also include GeO_2_ glass for which both Brillouin and Raman spectroscopic data are available^36,42^. This allows the incursion into an exotic chemical space of glass-forming melts and thus further test our findings.

The derived ratio of elastic moduli $K/G$ ranges between 1.08 for pure SiO_2_ provided by Novikov et al.^36^ and 1.90 for diopside (Di, SiO_2_ ~ 50 mol%) measured here. We obtained a linear correlation ($\text{R}$^2^ = 0.94) between the $K/G$ ratio and SiO_2_ content (mol%) when all samples are considered:

4 $$\frac{K}{G}=-0.0146\left(8\right)\cdot {\text{SiO}}_{2}+2.50\left(6\right)$$

It is worth noting that an inspection of Fig. 1a reveals that the SiO_2_ from Zanatta et al.^39^ exhibits $K/G$ = 1.18, which slightly deviates from the linear relationship. We argue that such variability of $K/G$ may stem from the dramatic structural effect of impurities (e.g., ppm of OH^−^) on the measured properties of nominally fully polymerized SiO_2_ glass^43,44^. Here, we consider the $K/G$ from Zanatta et al.^39^ because they provide Brillouin velocities and boson peak positions measured from the same sample (Spectrosil).

The BP dominates the low-frequency region of the Raman spectra of glasses and the peak position ${\omega }_{BP}$ can be retrieved by fitting the low-frequency part of the cross-polarized Raman spectra with a log-normal function^45^. We describe the fitting procedure in “Materials, literature data and methods” section and show Raman spectra in Fig. S1. The derived and literature ${\omega }_{BP}$ values are listed in Table 2. Figure 1b shows ${\omega }_{BP}$ as a function of SiO_2_ content (mol%). With decreasing SiO_2_ content from pure silica to 50 mol%, ${\omega }_{BP}$ increases from 47.8 cm^−1^ (RhE) to 90.0 cm^−1^ (Di). Although the data are scattered, the peak position appears to follow a linear trend with SiO_2_ content ($\text{R}$^2^ = 0.88) given by the relation:

5 $${\omega }_{BP}=-1.0\left(1\right)\cdot {\text{SiO}}_{2}+133\left(7\right)$$

## Discussion

**Figure 2:**
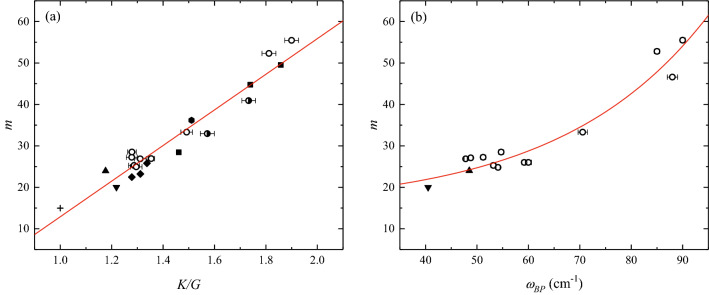
Melt fragility as a function of spectroscopic parameters of glass. (**a**) Relationship between the melt fragility *m* and ratio of the bulk and shear moduli $K/G$ of anhydrous samples. The *m* parameter is derived by fitting anhydrous melt viscosity data from the literature with Eq. (1) assuming ${\eta }_{\infty }=$ 10^−2.93^ Pa s, whilst $K/G$ is calculated using Eq. (3) and Brillouin velocities from this study and the literature. The two half-filled symbols indicate samples (Str and MSA) for which viscosity data used to derive $m$ and Brillouin data from this study used to obtain $K/G$, are derived from different samples (see “Literature data” paragraph for details). The red line represents the linear fit of the data (Eq. 6). Samples used here are seven rhyolites (Rh series), MSA, Str, DGG-1, An, Di, SiO_2_, GeO_2_, And, Bas, Phon, Foid, HPG8_Na05, HPG8_K05, HPG8_Li05 and the synthetic sample (cross, see Literature data paragraph for references). Literature data in (**a**) are: (square) Whittington et al.^35^, (hexagon) Richet and Polian^40^, (diamond) Hushur et al.^41^, (solid upward triangle) Zanatta et al.^39^, (solid downward triangles), Novikov et al.^36^. (**b**) Relationship between the melt fragility $m$ and ${\omega }_{BP}$ position of anhydrous samples. The BP position is derived by fitting the low-frequency Raman spectra of the glasses using a log-normal equation (see “Materials, literature data and methods” paragraph for details). The red line represents the exponential fit of the data (Eq. 7). Samples used here are seven rhyolites (Rh series), DGG-1, An, Crd, Di, HO, SiO_2_ and GeO_2_. The chemical composition and data sources are listed in Tables 1 and 2, respectively. Literature data in (**b**) are: (solid upward triangle) Zanatta et al.^39^, (solid downward triangle) Zanatta et al.^42^.

The possibility of deriving melt viscosity from the spectroscopic analysis of their parental glasses first relies on the identification of a relationship between the melt fragility *m* and glass transition temperature ${T}_{g}$ (Eq. 1) with at least one of the Brillouin- and Raman-derived parameters, namely the ratio of the bulk and shear moduli $K/G$ and ${\omega }_{BP}$. This can be addressed with anhydrous melts for which a reliable estimation of $m$ and ${T}_{g}$ can be achieved by combining high- and low-viscosity data measured by micropenetration, parallel plate and concentric cylinder viscometry. We collected anhydrous viscosity data (*N* = 468) from the literature (Table 2) and use the MYEGA formulation (Eq. 1) assuming ${\eta }_{\infty }={10}^{-2.93}$ Pa s (see Ref.^64^) to derive $m$ and ${T}_{g}$ (Table 2) of anhydrous melts. We then compare the derived fitting parameters with Brillouin and Raman data. Importantly, five anhydrous melts were excluded from the comparison in Fig. 2 (RhB, Teph and Trach for the Brillouin approach, and RhB, MSA and Str for the boson peak approach) because these samples were isolated to externally validate our approach together with all of the hydrous data. We find significant correlations between the Brillouin (Fig. 2a) and Raman (Fig. 2b) data with melt fragility $m$ over the entire chemical space explored in our study. A linear correlation (Fig. 2a) describes the relationship between melt fragility $m$ and $K/G$ ratio ($\text{R}$^2^ = 0.93):

6 $$m=43\left(3\right)\cdot \frac{K}{G}-31\left(4\right).$$

When volcanologically relevant (i.e. multicomponent) melts are considered, the SiO_2_-rich calcalkaline rhyolites^10^ and haplogranite HPG8 melts^41^ exhibit the lowest $K/G$ (~ 1.30) and $m$ (22.5–28.5), the SiO_2_-poor Foid sample^35^ shows the highest $K/G$ (1.86) and fragility (49.5), whereas samples with an intermediate SiO_2_ content (Phon, And, Str, Bas)^35,40^ are in between. Literature data of simple systems (pure SiO_2_ and GeO_2_)^36,39^ further extend the correlation to lower values of both $K/G$ and $m$. The anorthite and diopside samples^20^ exhibit $K/G$ equal to 1.81 and 1.90, respectively. Overall, our results agree with those of Novikov et al.^36^ who showed a linear correlation between $m$ and $K/G$ for simple glass-forming melts within a large interval of $m$ (20 $\lesssim m\lesssim$ 100). We derived $m$ of the multicomponent systems considered in this study and found that the Novikov et al.^36^ model can predict the melt fragility $m$ of strong systems, whereas a deviation is observed when more fragile systems are considered. For instance, we calculated (Eq. 1) $m$ = 20 for the strong GeO_2_ and derived $m$ = 22.1 with Eq. (6), and the remarkable literature^36^ prediction is *m* = 23.4. For more fragile systems such as the Foid sample, Eq. (1), provides *m* = 49.5 and Eq. (6) *m* = 49.7, whereas the estimation from the literature^36^ yields *m* = 42. This deviation can be attributed to: (1) the substantially different (i.e. multicomponent) and more restricted (i.e. dominantly SiO_2_-bearing) compositional space explored in this study; and (2) the assumed ${\eta }_{\infty }$ value that here is ${10}^{-2.93}$ Pa s while in Novikov et al.^36^ appears to be ${10}^{-4}\,\text{Pa s}$. Although these differences in the estimate of $m$ may be dictated by the chemical domain and strategy used to fit the viscosity data (Vogel–Fulcher–Tammann (VFT) and MYEGA equations and associated ${\eta }_{\infty }$), our results (Fig. 2a,b) fully confirm the literature observations^31,36^.

Similar to the BLS results, Fig. 2b shows a simple correlation between the fragility and BP position ${\omega }_{BP}$. We find that the correlation between ${\omega }_{BP}$ and $m$ can be empirically described via the following exponential function (${\text{R}}^{2}$ = 0.94):

7 $$m=1.7\left(4\right)\text{exp}\left({\omega }_{BP}/28\left(4\right)\right)+14.97,$$

where 14.97 is the minimum possible $m$ value^25,64^ within the MYEGA formulation (Eq. 1) adopted here (see “Literature data” paragraph for details). With decreasing melt fragility, the boson peak position shifts from approximately 48 cm^−1^ (pure SiO_2_) to 90 cm^−1^ (Di sample). From a fundamental perspective, our BLS (Fig. 2a) and RS (Fig. 2b) results point out the dominant role of acoustic modes in the BP region (e.g. Refs.^39,65^).

**Figure 3:**
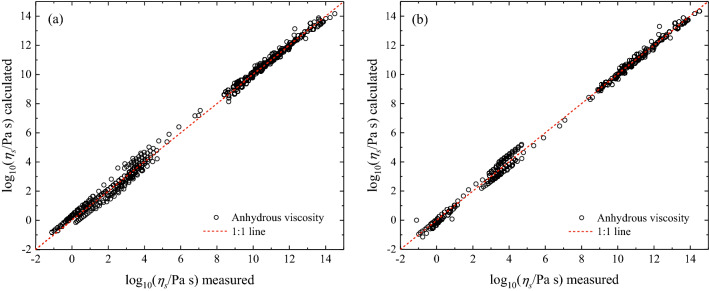
Predictions of anhydrous viscosity used for model calibration. (**a**) Comparison between anhydrous viscosity data (*N*_*dry*_ = 441) from the literature and MYEGA predictions (Eq. 1, where ${\eta }_{\infty }=$ 10^−2.93^ Pa s) using the melt fragility *m* derived by Brillouin data (Table 2) $m$ via Eq. (6). (**b**) Comparison between anhydrous viscosity data (${N}_{dry}=$ 288) from the literature and the model predictions (Eq. 1) using the melt fragility $m$ derived by ${\omega }_{BP}$ via Eq. (7).

The Brillouin and Raman data suggest that the spectroscopic analysis enables the estimation of the melt fragility, and glasses can therefore be used to estimate the viscosity of their parental melts, provided ${T}_{g}$ is known. The glass transition temperature ${T}_{g}$, which is the temperature at which the viscosity is 10^12^ Pa s, can be derived via DSC measurements using a small amount of glass (~ 10 mg) when subjected to a specific thermal treatment. In this measurement, the relaxed liquid is first cooled into the supercooled region at 10 K min^−1^ and the following DSC heating scan is performed at the same rate. With the adoption of this protocol, the onset of the glass transition corresponds to ${T}_{g}$ (see Refs.^20,24^ and references therein for a review). This procedure excludes the need to measure the viscosity to obtain ${T}_{g}$. Therefore, in order to retrieve the viscosity of melts from spectroscopic measurements of glasses, one needs to estimate $m$ by either Brillouin or Raman spectroscopy and measure ${T}_{g}$ via DSC. Figure 3a,b compare the measured viscosity from the literature and our prediction with the MYEGA equation (Eq. 1), assuming ${\eta }_{\infty }=$ 10^−2.93^ Pa s, using ${T}_{g}$ listed in Table 2 and *m* derived via Brillouin velocities with Eq. (6) (Fig. 3a) and Raman spectroscopy using Eq. (7) (Fig. 3b). The Brillouin and Raman models have a root-mean-square-error (RMSE) of 0.26 and 0.27 log units, respectively.

Finally, we use an external dataset to validate our approach. We explored the largest possible chemical space of volcanologically-relevant glasses for which viscosity and spectroscopic data are available for the same sample. We collected a set of anhydrous and hydrous melts whose viscosity was independently measured over ~ 14 orders of magnitudes as well as Brillouin and Raman data of their parental glasses. The estimated viscosity ${\eta }_{s}\left(T\right)$ was calculated using Eq. (1) with ${\eta }_{\infty }=$ 10^−2.93^ Pa s and ${T}_{g}$ from the literature (see Table 2), whereas the melt fragility *m* is Brillouin-derived, i.e. calculated using Eq. (6) and the $K/G$ ratio obtained from the sound velocities measured by BLS, or Raman-derived, obtained via Eq. (7) and the BP position ${\omega }_{BP}$ measured from RS.

**Figure 4:**
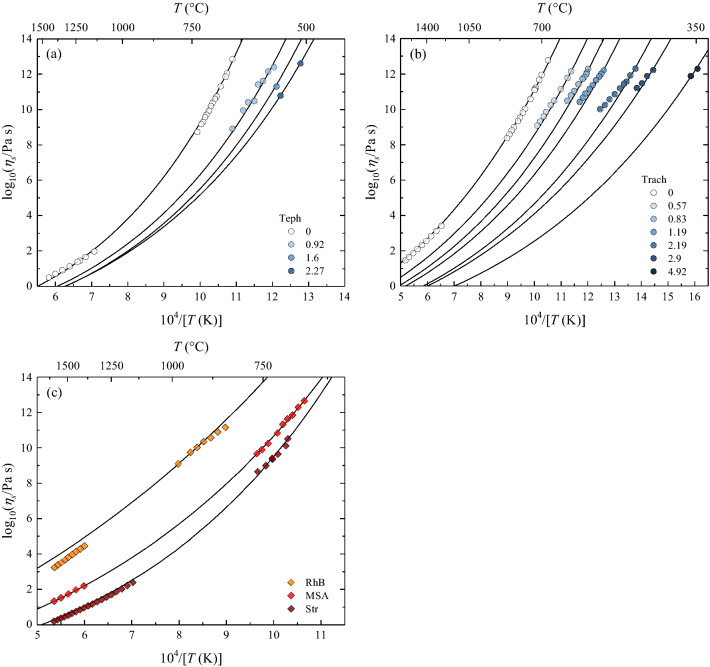
External predictions of anhydrous and hydrous viscosity. (**a**) Measured (symbols) anhydrous and hydrous viscosity data for tephrite (Teph)^61^ and predictions (lines) using Brillouin velocities (Table 2). Numbers in the legend indicate the water content of samples in wt%. (**b**) Measured (symbols) anhydrous and hydrous viscosity data for trachyte (Trach)^11^ and predictions (lines) using Brillouin sound velocities (Table 2). Numbers in the legend indicate the water content of samples in wt%. (**c**) Measured (symbols) anhydrous viscosity data for the calcalkaline rhyolite RhB^10^, MSA andesite^37,49^ and Str basalt^50,51^. Lines are predictions using the boson peak position (Table 2) derived by Raman spectroscopy.

Brillouin data (Table 2) of anhydrous and hydrous systems (Bas, Teph, Foid, Trach, Phon, And, RhB) were used to validate our approach using viscosity data from the literature (${N}_{validation}=$ 198)^10,11,17,59–61^. Furthermore, we used $K/G$ = 1.16 for the HPFS 7980 fused silica (SiO_2_, OH^−^ content between 800 and 1000 ppm) to calculate the viscosity at 1585 °C, which corresponds^63^ to the softening point of the sample (${\eta }_{s}=$ 10^6.6^ Pa s). In Fig. 4a,b, we report two examples for the relatively SiO_2_-poor and SiO_2_-rich volcanic Teph and Trach samples, respectively. Figure S4 shows the remaining samples (And, Bas, Foid, Phon and RhB). Moreover, Fig. 4c compares the anhydrous measured viscosity (${N}_{validation}=$ 57) and Raman-based predictions using the BP position of the basalt (Str, viscosity data from Refs.^50,51^), andesite (MSA; viscosity data from Ref.^49^) and rhyolite (RhB; viscosity data from Ref.^10^) glasses. An inspection of the results for anhydrous viscosities in Fig. 4a–c notably demonstrates that our approach provides accurate low- and especially high-temperature projection (lines) of viscosity, as demonstrated by the remarkable prediction of the water-free viscosity in the low-viscosity regime (${{\eta }}_{s}<$ 10^4^ Pa s). Here, we observe that the BLS approach (Fig. 4a,b) perform slightly better than the RS approach (Fig. 4c). Concerning hydrous samples and based on BLS data from Ref.^35^, Fig. 4a shows that we can successfully predict the measured viscosity^61^ as a function of temperature for SiO_2_-poor tephritic glasses (Teph) characterized by 0.92, 1.6 and 2.27 wt% H_2_O. We include in our comparison (Fig. 4a) the prediction of ${\eta }_{s}\left(T\right)$ for those samples whose viscosity did not suggest crystallization and/or water loss during the measurement, as discussed in Ref.^61^. We further validate our Brillouin-based approach with hydrous SiO_2_-rich trachyte (Trach) melts for which both viscosity and Brillouin data are known^11,35^. Figure 4b illustrates that our approach accurately predicts anhydrous viscosities over ~ 12 orders of magnitudes, similar to the Teph anhydrous sample (Fig. 4a). However, a careful inspection of the results reveals that a slightly different picture is depicted.

Our approach can indeed accurately predict the measured viscosity of relatively water-poor samples (H_2_O ≤ 0.83 wt%) within the interval of 10^9^ $<{\eta }_{s}<$ 10^12^ Pa s. For the water-rich samples (H_2_O ≥ 1.19 wt%), the accurate prediction is limited to ${\eta }_{s}$ ~ 10^11^ Pa s because at lower viscosities, our approach increasingly underestimates the viscosity with increasing temperature. This is due to the opposite behaviour observed for the melt fragility as a function of water content when our $m$ estimates are compared with those provided by the literature^11^, calculated using viscosity data and the VFT description of ${\eta }_{s}\left(T\right)$ in Ref.^11^. The Brillouin-derived $m$ increases from 30.9 (H_2_O = 0 wt%) to 36.8 (H_2_O = 4.92 wt%), whereas the literature-derived $m$ decreases with H_2_O content from 32.9 to 26.3. A separate study is required to experimentally investigate this aspect through, for instance, the combination of Raman spectroscopy and TEM imaging^20,23,66^ to analyse glasses before and after viscosity measurements; thus we discuss the expected fragility behaviour with H_2_O for trachytic melts based on glass structure and thermodynamics concepts.

**Figure 5:**
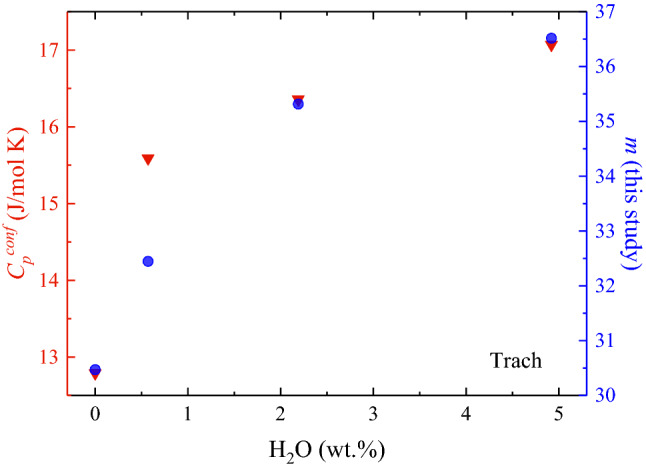
Configurational heat capacity ${C}_{p}^{conf}$ (triangles, data from Ref.^68^) and the Brillouin-derived melt fragility *m* (circles, Table 2) of hydrous trachyte (Trach) as a function of water content. The increase in ${C}_{p}^{conf}$ with H_2_O suggests that the addition of water depolymerizes the melt structure, which is in turn expected to increase the melt fragility. Our Brillouin-derived $m$ values therefore agree with the expected scenario.

Melt fragility is a kinetic property of glass-forming melts that positively correlates with several chemical and thermodynamic properties of glasses^67–73^ such as the degree of structural polymerization and especially the configurational heat capacity (${C}_{p}^{conf}$) at the glass transition temperature^67,72,74,75^. The key aspect we address here is that strong and polymerized melts are characterized by low ${C}_{p}^{conf}$, whereas fragile and depolymerized melts exhibit high ${C}_{p}^{conf}$. Although water depolymerizes the structure of silicate melts, a paradox emerges^68,74,76,77^ when ${C}_{p}^{conf}$ data of hydrous glasses are compared with $m$ derived by the empirical fit of viscosity data (e.g., VFT equation)^78,79^. Indeed, heat capacity measurements^68^ of relatively polymerized systems such as the Trach sample discussed here show, as expected, that ${C}_{p}^{conf}$ increases with increasing H_2_O content, whereas $m$ derived by the VFT fit decreases with water content^11^. Figure 5 shows the water dependence of the measured ${C}_{p}^{conf}$ (data from Ref.^68^) and calculated $m$ (this study, via Brillouin velocities) for the Trach samples.

The relationship illustrated in Fig. 5 shows that ${C}_{p}^{conf}$ and $m$ similarly increase with increasing water content. This observation agrees with the idea that water depolymerizes the structure of relatively polymerized systems, and the Brillouin-derived increase of $m$ with water content therefore agrees with the expected scenario^67,75^ based on the independent measure of ${C}_{p}^{conf}$ on the same samples^68^: namely $m$ increases with the depolymerization of the melt structure. An attempt to reconcile the contradictory aspects listed so far may consider that hydrous samples subjected to viscosity measurement above ${T}_{g}$ (Fig. 3a) underwent a subtle but significant textural and/or chemical modification (i.e. water loss) that gradually became increasingly severe with increasing experimental temperature and/or water content. Such a modification eventually resulted in a slight increase in viscosity, which led to an apparent decrease of $m$ with water content. However, we point out that our interpretation is highly speculative, and our result rather offers a hypothesis for future studies to address the link between ${C}_{p}^{conf}$ and viscosity that notably remains an outstanding problem for modelling the melt viscosity from a physical standpoint^67,75^.

**Figure 6:**
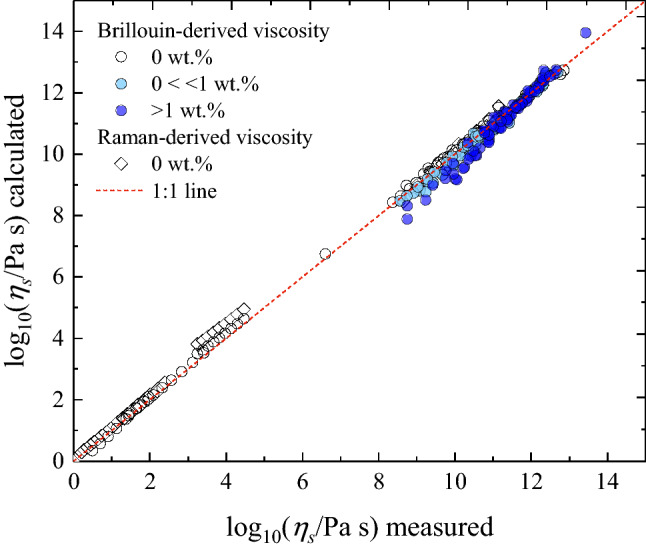
Comparison between measured viscosity values with Brillouin-derived (circles) and Raman-derived (empty diamonds) values. For the Brillouin-derived viscosity, both anhydrous (empty circles) and hydrous (coloured circles) data are reported. Hydrous data are grouped according to their water content (< 1 and > 1 wt%). The largest deviation between the measured and calculated values is observed for samples characterized by both the highest water content and lowest measured viscosity (see text for details). Only anhydrous viscosity is available for the Raman-derived data.

Figure 6 summarizes the results of the external validation of our Brillouin- and Raman-based approach to derive ${\eta }_{s}\left(T\right)$ of anhydrous and hydrous melts over ~ 14 orders of magnitudes using 278 viscosity data (RhB, Bas, Str, And, MSA, Teph, Foid, Trach, Phon, HPFS 7980 fused silica). Samples are coloured according to water content. The largest deviation between the measured and calculated viscosity values is observed for the Phon melts with H_2_O > 1 wt%. Here, as discussed for the Trach system, the derived $m$ via Brillouin velocities (Table 2) and measured^68^ ${C}_{p}^{conf}$ increase with increasing H_2_O, whereas the $m$ values derived from the fit of the viscosity data decreases^11^. The Brillouin-derived *m* values for the And, Bas and Foid samples vary slightly (Table 2) with water content. All in all, our BLS and RS-based approaches can predict the viscosity of anhydrous and hydrous volcanologically-relevant melt with a RMSE of 0.24 log units (BLS) and 0.30 log units (RS).

Finally, we stress that the glasses used and considered here for deriving the BLS and RS data were subjected to virtually unknown and variable cooling rates. Nevertheless, the success of our validation suggests that typical laboratory cooling rates do not significantly affect the relationship between $K/G$ and ${\omega }_{BP}$ of the glasses and melt fragility $m$ of their parental melts within the chemical space explored here. This is in line with observations from (1) Whittington et al.^35^ who subjected unrelaxed and relaxed glasses to BLS (see “Brillouin spectroscopy” paragraph for more details) and (2) McIntosh et al.^80^ who studied the ${\omega }_{BP}$ of alkali silicate glasses. Both studies found little difference in the measured vibrational properties between quenched and annealed glasses. However, a more robust dataset is needed to investigate this carefully and we are confident that our study will stimulate new studies in this direction.

## Conclusions and implications

We introduce a novel approach to derive the viscosity of volcanic and technical glass-forming melts covering over ~ 14 orders of magnitudes. This approach transcends the need to perform viscosity measurements and is based on (1) the MYEGA formulation^25^ of melt viscosity over temperature, (2) Brillouin^31^ or Raman analysis of glasses at ambient conditions to retrieve the melt fragility $m$ via the ratio of elastic moduli $K/G$ or the boson peak position ${\omega }_{BP}$ and (3) differential scanning calorimetry^20,81^ for measuring the glass transition temperature ${T}_{g}$. The model was trained and validated at volcanologically relevant conditions using two large and non-overlapping anhydrous and hydrous datasets, encompassing virtually the entire range of magmatism on Earth. The complementarity of the Brillouin- and Raman-derived results extends the possibility of applying our approach with small amounts of sample (~ 10 mg of glass) using standard laboratory equipment and ensures cross-validation of the results. The possibility of deriving the viscosity of homogenous and nanocrystal-free volcanic melts enables the accurate quantification of the effect of nanocrystal formation on magma viscosity and therefore the eruptive dynamics of volcanoes. Finally, our results support the link between acoustic modes and the boson peak, which provides further insight into its long-debated nature.

## Materials, literature data and methods

### Materials

We subjected 16 anhydrous glasses to Raman and Brillouin spectroscopic analyses. Table 1 lists the chemistry of the tested samples. Eight samples consist of synthetic iron-bearing calcalkaline rhyolitic glasses (Rh series) used for viscometry by Di Genova et al.^10^. We also used four natural and iron-bearing glasses obtained by melting volcanic rocks: a dacite^38^ (HO) from Mt. Fuji volcano (Japan); an andesite^37^ (MSA) from Montserrat; a trachybasalt^20^ (Etn) from Mt. Etna volcano (Italy) and a basalt from Stromboli volcano (Str, Italy). Four iron-free glasses from Di Genova et al.^20^ were also selected: two fully polymerized glasses (anorthite An and cordierite Crd) and two depolymerized glasses (standard glass DGG-1 and diopside Di).

### Literature data

The viscosity data for the iron-bearing calcalkaline rhyolitic samples are provided by Di Genova et al.^10^, whilst for MSA we used data from Neuville et al.^49^ who measured the viscosity of an andesite melt (SiO_2_ 61.2 wt%) chemically equivalent with our MSA sample (Tab. 2). Giordano et al.^50^ and Misiti et al.^51^ measured the viscosity of the Str basalt. The glass transition temperature of the Etn trachybasalt glass was measured by Di Genova et al.^20^. The low-temperature viscosity data for the standard glass DGG-1, diopside (Di), anorthite (An) and cordierite (Crd) are provided by Di Genova et al.^20^ and Al-Mukadam et al.^55^. The high-temperature viscosity data for these systems are listed elsewhere^46,52–54,56–58^. No nanocrystal-free melt viscosity data are available for Etn^20^ and dacite (HO) melts. The viscosity data for SiO_2_ are those from Urbain et al.^46^ and Bucaro and Dardy^82^, whilst for GeO_2_ are provided by Napolitano and Macedo^47^ for the high-viscosity regime and by Sharma et al.^48^ for the low-viscosity regime.

We drew on the Brillouin spectroscopy literature of multicomponent systems of volcanological interest. Table 2 lists the Brillouin data for those glasses whose ${\eta }_{s}\left(T\right)$ of the corresponding liquid is known. The Brillouin and viscosity data of anhydrous peralkaline haplogranite glasses (HPG8_Li05, HPG8_Na05, HPG8_K05) are provided by Hushur et al.^41^ and Hess et al.^62^, respectively. For these samples, it was possible to derive $K/G$ both at ambient temperature and ${T}_{g}$. We found that the $K/G$ increses from 1.34 to 1.40 and from 1.31 to 1.35 for HPG8_Li05 and HPG8_Na05, respectively. Richet and Polian^40^ and Richet et al.^17^ measured Brillouin velocities and ${\eta }_{s}\left(T\right)$ for anhydrous and hydrous iron-free andesitic glasses (And). Richet and Polian^40^ measured Brillouin velocities from compacted and relaxed glasses. We use the data obtained from the relaxed samples because a complete dataset for both dry and hydrous glasses is provided. Whittington et al.^11,35^, Robert et al.^60^ and Robert^59^ reported Brillouin and viscosity data for anhydrous and hydrous iron-free phonolite (Phon), basalt (Bas) and foidite (Foid) systems. We calculated the average velocities for each sample when more measurements on the same sample were performed. We also include Brillouin velocities (Novikov et al.^36^ and references therein) of fully-polymerized glass-forming systems such as SiO_2_ and GeO_2_. We constrained the relationship between the melt fragility $m$ and Brillouin-derived elastic moduli, bulk (*K*) and shear (*G*), over the largest landscape possible by introducing a synthetic and perfectly strong sample (Synth) characterized by $m$ = 14.97 and $K/G=$ 1^36^. The fragility value was derived by previous studies^25,64^ that used the MYEGA formulation for ${\eta }_{s}\left(T\right)$ (Eq. 1) and the assumption that ${\eta }_{\infty }={10}^{-2.93}$ Pa s (see Ref.^64^) on which our study is based. Yet simultaneously, the $K/G$ ratio (see “Brillouin spectroscopy” paragraph below) for strong glasses is not expected to be lower than 1^36^. Finally, the boson peak position ${\omega }_{BP}$ of SiO_2_ and GeO_2_ glasses is given by Zanatta et al.^39,42^.

### Brillouin spectroscopy

Brillouin spectroscopy analysis of 50-μm-thick and double-sided polished glasses was carried out in platelet geometry at ambient condition using a solid-state Nd:YVO4 laser source with a wavelength of ${\lambda}=$ 532 nm and power at the source of 50 mW. The Brillouin frequency shift was quantified using a six-pass Fabry–Perot interferometer^83^ combined with a single-pixel photon counter detector. The measurements were conducted using a symmetric forward scattering geometry^84,85^ with a scattering angle ${\theta }=$ 79.8°. The scattering angle was calibrated using a silica reference glass. Experimentally determined frequency shifts (${\Delta }{\omega }$) were converted to longitudinal (${v}_{p}$) and shear (${v}_{s}$) sound velocities according to the equation:

8 $${v}=\frac{{\Delta }{\omega }{\lambda }}{2sin\left({\theta }/2\right)},$$

where *λ* is the laser wavelength in air. We explored the potential effect of deviation from symmetric platelet geometry due to possible minor sample tilting on the measured velocities by collecting between 8 and 9 spectra for each sample at different rotation angle ${{\rm X}}$ (from − 180 to + 180°). Longitudinal and shear sound velocities did not have any systematic variation due to the sample tilting, the minor deviations from the average values are accounted for the reported uncertainties.

Finally, we explored whether or not the cooling rate affects the derived $K/G$ ratio. Whittington et al.^35^ subjected two water-bearing basalts (H_2_O = 1.39 and 3.01 wt%, respectively) to Brillouin measurements. They used both unrelaxed and relaxed samples for each water content, and we found that $K/G$ does not change: the ratio is 1.80 for both unrelaxed and relaxed samples with H_2_O = 1.39 wt%, and $K/G$ = 1.68 for both samples with H_2_O = 3.01 wt%. Furthermore, Richet and Polian^40^ provided Brillouin velocities from compacted and relaxed glasses. We did not find a significant variation between the $K/G$ of the compacted (1.57) and relaxed (1.58) sample in the case of an andesitic glass with 2.7 wt% H_2_O. We instead found a decrease of $K/G$ when the relaxed sample was measured (from 1.50 to 1.43 for H_2_O = 1 wt% and from 1.61 to 1.50 for H_2_O = 3.5 wt%). Here, we use the data obtained from the relaxed samples because a complete dataset for both dry and hydrous glasses is provided.

### Raman spectroscopy

Polarized Raman spectra were collected using a Horiba Jobin-Yvon T64000 triple spectrometer set in double subtractive/single configuration with three holographic gratings of 1800 lines/mm and equipped with a CCD detector (1024 × 256 pixels) cooled by liquid nitrogen. We used the 514.5 nm line of an Ar-Kr ion gas laser (Spectra Physics Satellite 2018 RM) as the excitation source. The measurements were carried out in backscattering geometry using a microscope with a 50× objective (numerical aperture = 0.50) to probe the sample and collect the scattered radiation. Samples were polished and optically and spectroscopically^38^ inspected before and after the measurements without observing alteration on the μm-length scale. Raman spectra were acquired in air at room temperature over the spectral region between 10 and 1300 cm^−1^ (Stokes side). Each spectrum was corrected by subtracting the rotational Raman spectrum of air in the low-wavenumber region below 180 cm^−1^, and a linear baseline due to the weak luminescence background. Following the effective relation proposed by Shuker and Gammon^86^ and considering the Stokes side of cross-polarized (HV) Raman spectra, the BP features can be obtained from the reduced Raman intensity ${I}^{red}\left({\omega }\right)$, namely:

9 $${I}^{red}\left({\omega }\right)=\frac{{I}^{exp}\left({\omega }\right)}{{\omega }\left[n\left({\omega },T\right)+1\right]}=\text{C}\left({\omega }\right)\frac{g\left({\omega }\right)}{{{\omega }}^{2}}.$$

In HV spectra, the low-wavenumber tail of the prominent spectral feature related to rocking and symmetric bending motions of bridging oxygen is suppressed, thus easing the determination of the BP spectral features. Here, we correct the experimental Raman intensity ${I}^{exp}\left({\omega }\right)$ for the thermal population of the vibrational modes given by the Bose–Einstein factor $n\left(\omega ,T\right)={\left[\text{exp}\left(\hbar \omega /{k}_{B}T\right)-1\right]}^{-1}$ where $\hbar$ and ${k}_{B}$ are the reduced-Planck and Boltzmann constants, respectively. The obtained spectrum is proportional to the reduced vibrational density of states $g\left(\omega \right)/{\omega }^{2}$ through the coupling function $C\left(\omega \right)$. Although the nature of this parameter is debated, much evidence indicates that it is linear in the BP region (Zanatta et al.^39^ and references therein). In this study, we consider only the BP position defined as the maximum in the reduced intensity and obtained from a fit with a log-normal function^45^:

10 $$I\left(\omega \right)\propto \text{exp}-\left\{{\left[\text{ln}\left(\omega /{\omega }_{BP}\right)\right]}^{2}/2{\sigma }^{2}\right\},$$

where $\sigma$ is the width of the BP.

## Supplementary Information

Supplementary Information 1.

## Data Availability

All data needed to evaluate the conclusions in the paper are present in the paper and/or the Supplementary Materials. Additional data related to this paper may be requested from the authors.
