# Supplementary material for: Estimating the viscosity of volcanic melts from the vibrational properties of their parental glasses
Source: Sci Rep. 2021 Jun 22;11:13072. doi: 10.1038/s41598-021-92407-5 (PMC8219844; doi:10.1038/s41598-021-92407-5)
Supplement: Supplementary file 1 — Supplementary Information 1. [file 41598_2021_92407_MOESM1_ESM.docx]

Supplementary information:

Estimating the viscosity of volcanic melts

from the vibrational properties of their parental glasses

Michele Cassetta^1^, Danilo Di Genova^2*^, Marco Zanatta^3^, Tiziana Boffa Ballaran^2^, Alexander Kurnosov^2^, Marco Giarola^4^, Gino Mariotto^1^

^1^Dipartimento di Informatica, Università di Verona, I-37123 Verona, Italy

^2^BGI, Bavarian Research Institute of Experimental Geochemistry and Geophysics, University of Bayreuth, D-95447 Bayreuth, Germany

^3^Dipartimento di Fisica, Università di Trento, I-38123, Trento, Italy

^4^CPT, Centro Piattaforme Tecnologiche, University of Verona, I-37134, Verona, Italy

*Corresponding author:

Danilo Di Genova: danilo.di-genova@uni-bayreuth.de

**Raman spectra**

Figures S1 and S3 show the low-frequency region of Raman spectra collected from SiO_2_-rich calcalkaline rhyolites (Rh series, Fig. S1), SiO_2_-intermediate multicomponent volcanic samples (HO, MSA, Etn, and Str Fig. S2), and iron-free samples (DGG-1, An, and Crd, Fig. S3). Spectra are shown after the subtraction of both rotational Raman spectra of the air and a linear weak luminescence background (see Materials section for details). The sample chemical composition is reported in Table 1, whereas the boson peak position is listed in Table 2.

**
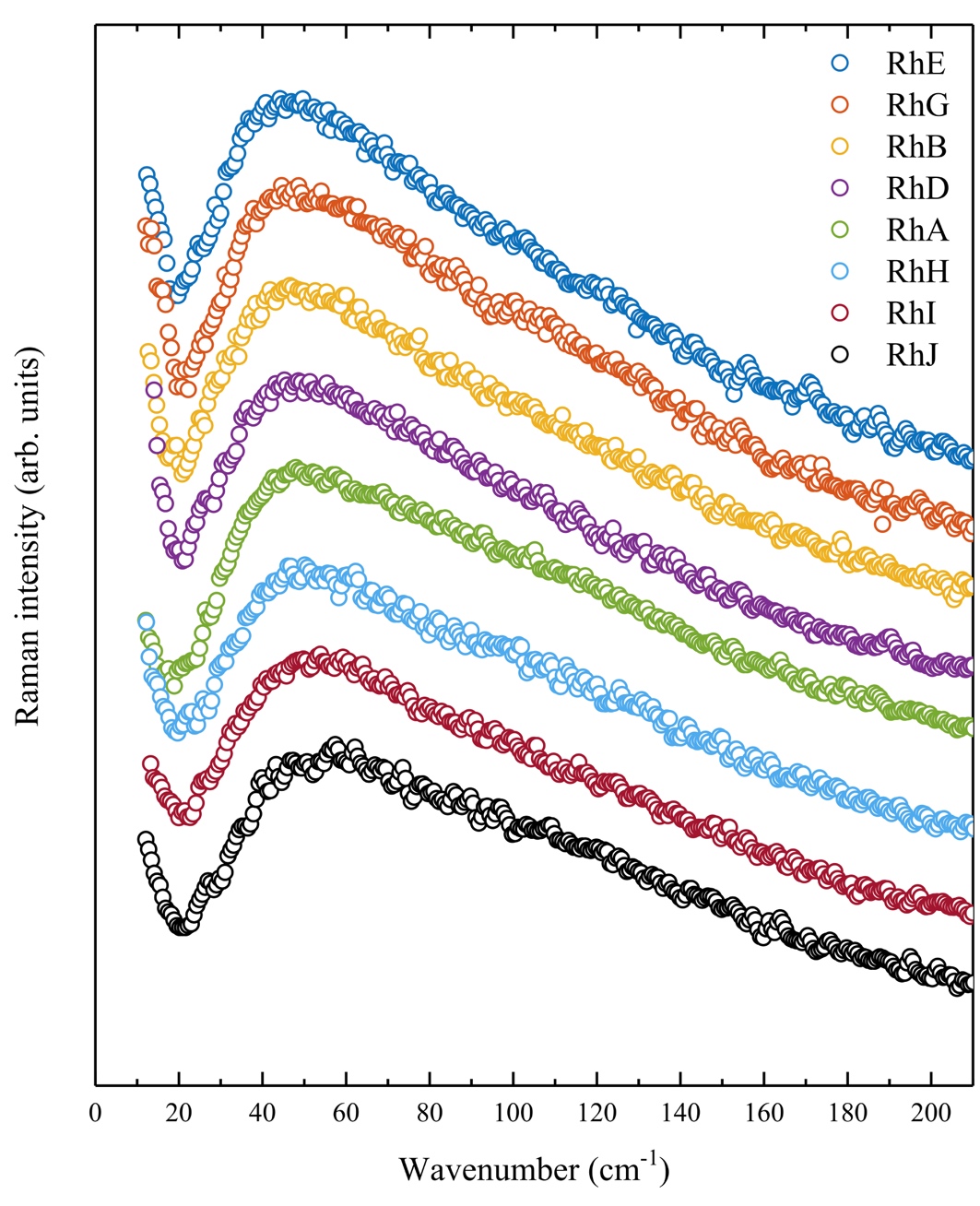
**

**Figure S1.** Low-frequency region of Raman spectra acquired from Rh rhyolites (Table 1). The spectra are shifted along the vertical axis.

**
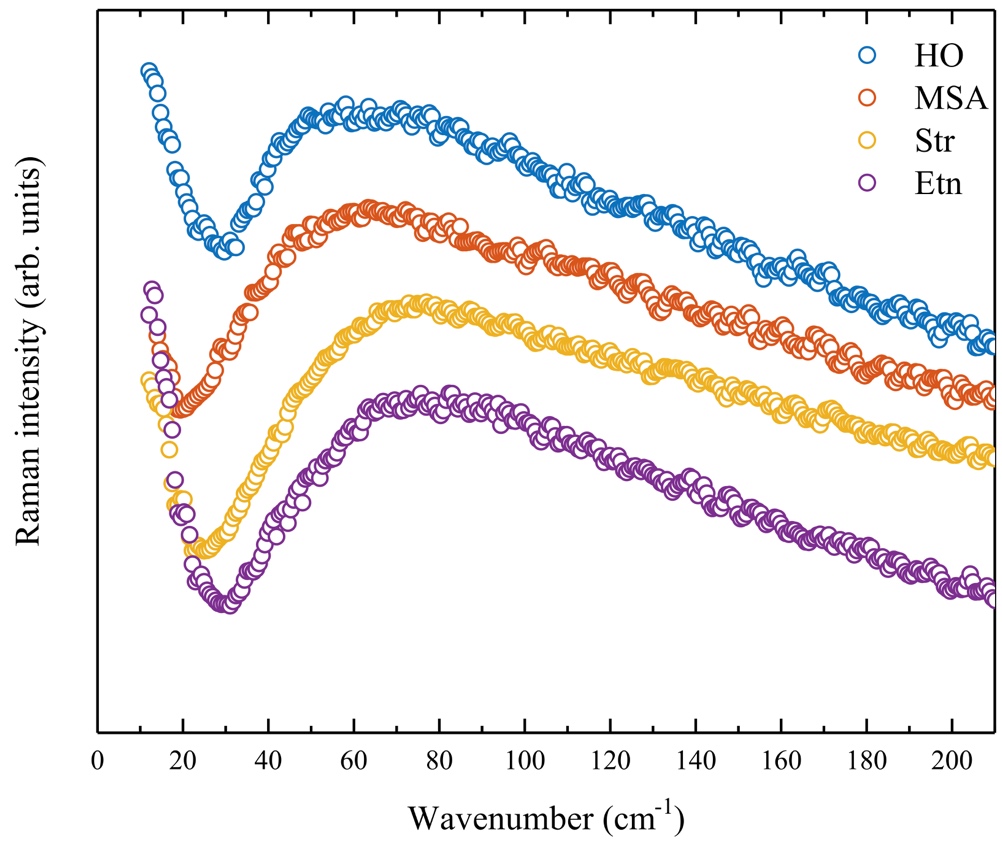
**

**Figure S2.** Low-frequency region of Raman spectra acquired from the HO, MSA, Str, and Etn samples (Table 1). Spectra are shifted along the vertical axis.

**
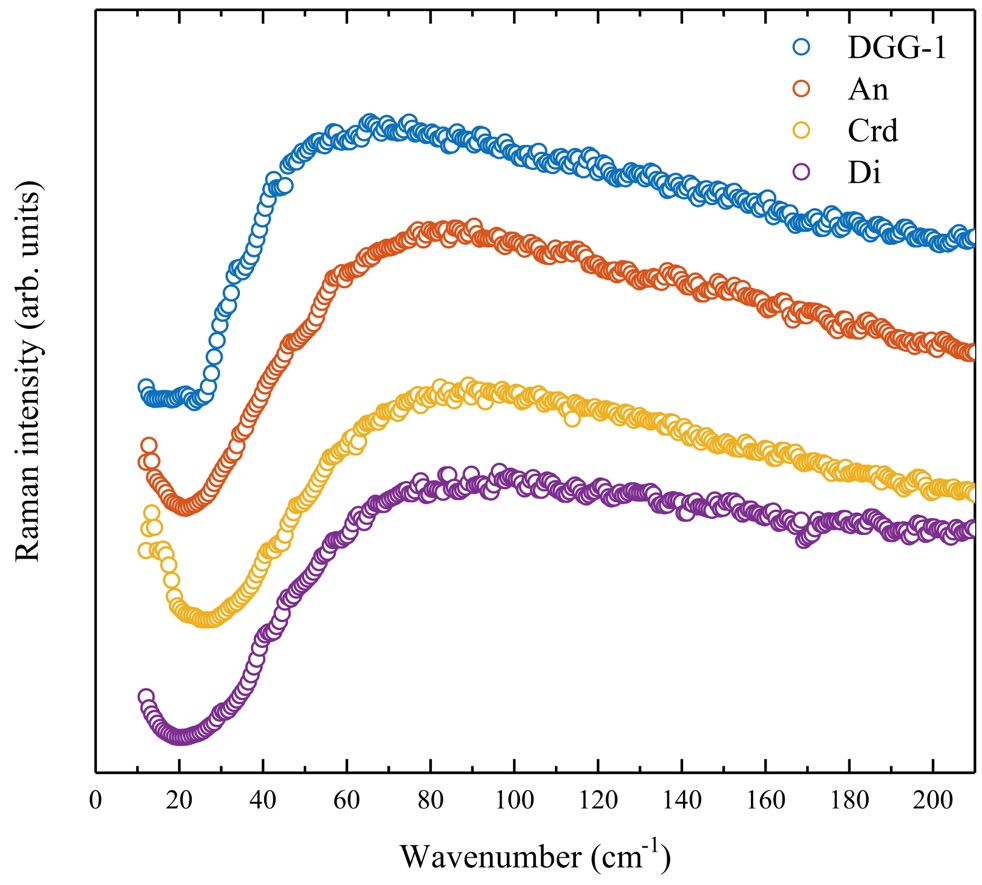
**

**Figure S3.** Low-frequency region of Raman spectra acquired from the iron-free samples DGG-1, An, Crd, and Di (see Tab. 1). Spectra are shifted along the vertical axis.

**External predictions of viscosity using BLS**

Figure S4 shows the measured (circles) and predicted (crosses) viscosities of the remaining samples used to externally validate the BLS model (Fig. 6).


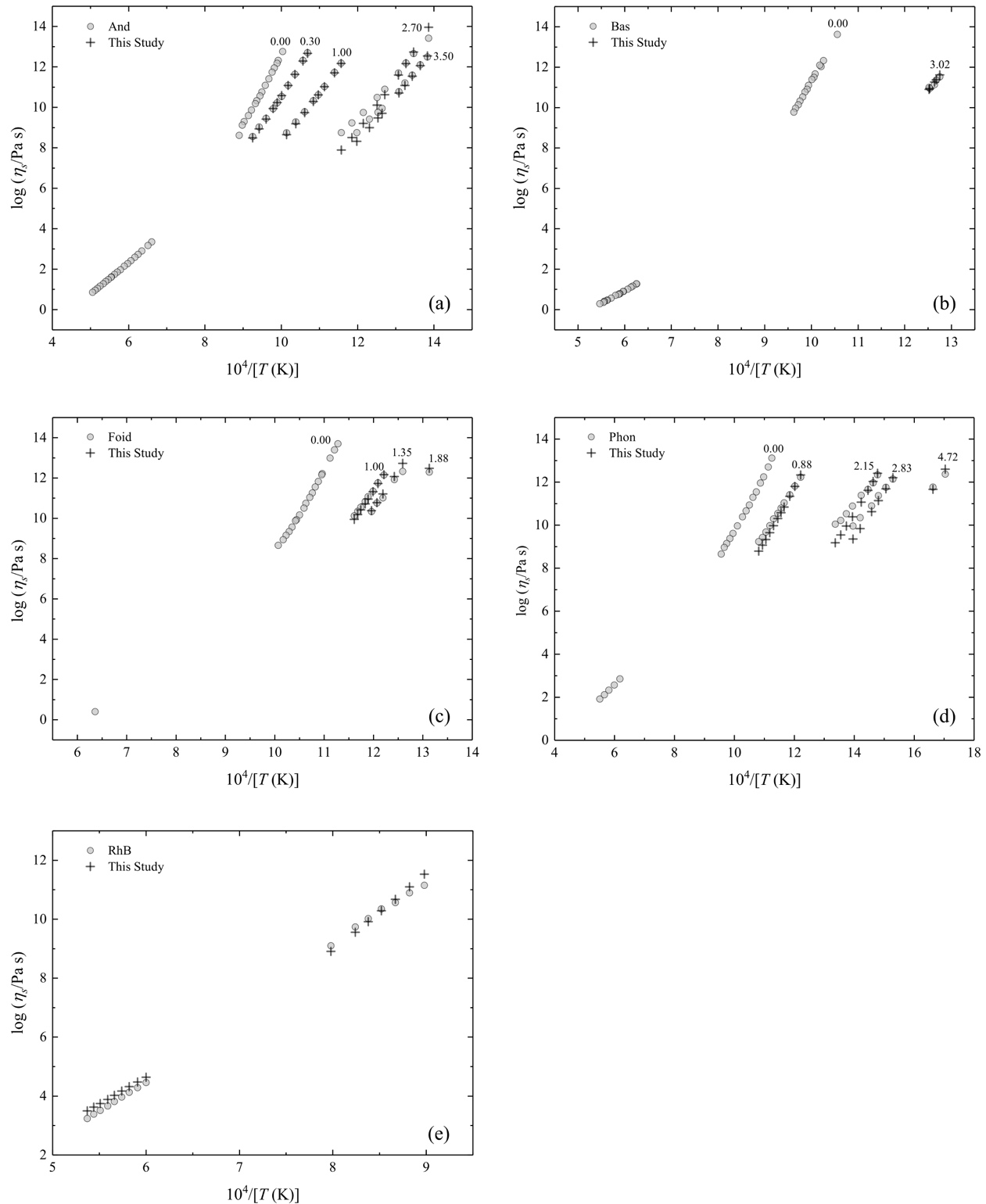


**Figure S4.** External predictions of anhydrous (only RhB) and hydrous viscosity. Measured (circles) viscosity data and predictions (crosses) using Brillouin velocities data for (a) And, (b) Bas, (c) Foid, (d) Phon and (e) rhyolite RhB. Labels indicate the water content of samples in wt%. Measured anhydrous viscosities for And, Bas, Foid and Phon samples were used to calibrate the BLS model (Fig. 3a) and are reported here as a benchmark for the hydrous viscosities.
